# May-Hegglin Anomaly (MHA): A Rare Platelet Disorder Presenting for Coronary Artery Bypass Grafting, Managed With Post-bypass Administration of Platelets and Desmopressin

**DOI:** 10.7759/cureus.93595

**Published:** 2025-09-30

**Authors:** Jonathan A Bond, Alec Statler, John Bozek, Mir Ali Abbas Khan, Matthew B Ellison

**Affiliations:** 1 Anesthesiology, West Virginia University, Morgantown, USA

**Keywords:** adult cardiac surgery, may-hegglin anomaly, myh-9 related disorder, platelets function, thromboelastography (teg), transfusion

## Abstract

May-Hegglin anomaly (MHA) is a hematologic disorder defined by large platelets (mean platelet volume >12.5 fL) and thrombocytopenia. While it remains unclear whether these patients exhibit clinical coagulopathy, bleeding complications may be confounded in those undergoing cardiac surgery requiring cardiopulmonary bypass (CPB). There are no current guidelines for the perioperative management of patients with MHA. Although some reports favor no treatment, limited case reports have described desmopressin (DDAVP) and platelet transfusion as additional strategies. Thromboelastography® with Platelet Mapping™ (TEG-PM) is one modality capable of monitoring coagulation, which can help guide intraoperative blood product transfusions, particularly in patients with underlying platelet disorders.

We report the successful management of a 70-year-old female with a history of MHA who presented for CABG and exhibited persistent bleeding despite adequate heparin reversal with protamine. A post-protamine TEG and TEG-PM were obtained. Prior to sternal closure, platelets were requested to treat ongoing bleeding. While the post-protamine TEG appeared normal, TEG-PM demonstrated platelet inhibition, prompting consideration of DDAVP. As a result, both platelets and DDAVP were administered, leading to hemostasis and cessation of bleeding.

Given the limited data, patients with MHA should undergo a thorough perioperative hematologic evaluation, including a history of prior bleeding episodes in addition to conventional laboratory studies. Although an increased risk of hemorrhage has not been definitively demonstrated, preparation with blood products and DDAVP should be considered when a heightened risk of surgical coagulopathy exists, such as during cardiac surgery. In our case, we utilized TEG-PM as an additional tool to evaluate hemostasis, which guided the combined use of platelets and DDAVP. While future studies are needed to assess the utility of TEG-PM in the MHA population, it remains important to consider all available technologies to evaluate potential coagulopathy on an individualized basis.

## Introduction

May-Hegglin anomaly (MHA) is a rare blood disorder infrequently encountered in the practice of anesthesiology. Due to dysfunction of the MYH-9 gene, hematologic sequelae include Döhle bodies within leukocytes, increased platelet size, and thrombocytopenia [1]. Despite these findings, platelet function is generally preserved. Of the few case reports available in the literature, patients with MHA have received a spectrum of perioperative treatment regimens, ranging from platelet transfusion [2] to no therapy [3] and desmopressin (DDAVP) [4]. To date, there are no established guidelines in the United States for the perioperative management of patients with MHA. Reports of such patients undergoing cardiac surgery are even rarer. Given the baseline risk of surgical bleeding and the known platelet dysfunction that occurs with cardiopulmonary bypass [5], additional coagulopathic concerns may arise when managing MHA during cardiac surgery, including coronary artery bypass grafting (CABG).

Thromboelastography with Platelet Mapping (TEG-PM, TEG®; Haemonetics, Braintree, MA, USA) is a hematologic test capable of providing a functional clotting assessment in real time, helping guide blood product administration. Here, we describe our management of a patient with MHA undergoing cardiac surgery, with TEG-PM incorporated into the intraoperative care plan.

This case report was previously presented as an abstract at the Society of Cardiovascular Anesthesiologists Annual Meeting on April 24, 2021.

## Case presentation

**Figure 1:**
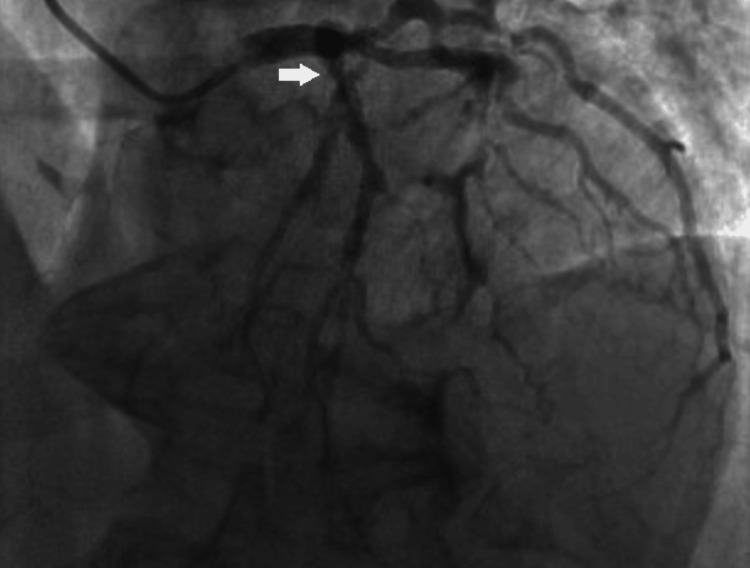
Left heart catheterization displaying in-stent stenosis of the left anterior descending coronary artery

A 70-year-old female with a history of smoking, type II diabetes mellitus, hypertension, hyperlipidemia, coronary artery disease (CAD) with remote percutaneous coronary intervention (PCI), and MHA was transferred to our institution for planned CABG. The patient had a positive stress test, and subsequent coronary angiography showed extensive in-stent stenosis of the left anterior descending (LAD) coronary artery (Figure 1), as well as a chronically occluded right coronary artery (RCA). A preoperative transthoracic echocardiogram was negative for systolic dysfunction (including regional wall motion abnormalities) and valvular pathology. The ejection fraction was preserved at 69%, with concentric hypertrophy as the only noteworthy finding. Specific to her history of MHA, the patient denied any previous episodes of bleeding and reported annual hematology follow-up for surveillance. She also denied any history of anesthetic or hemorrhagic complications with prior surgeries. Significant preoperative laboratory values included a platelet count of 93 × 10³/µL (reference: 150-400 × 10³/µL), morphology displaying large platelets, and a mean platelet volume of 12.8 fL (reference: 8.7-12.5 fL). The patient’s preoperative hemoglobin was 11.4 g/dL (reference: 11.5-16 g/dL).

A preoperative arterial line for continuous hemodynamic monitoring was placed prior to induction of general anesthesia. General endotracheal anesthesia was achieved uneventfully. A bolus of 10 grams of aminocaproic acid was administered after sternotomy, followed by a continuous infusion (1 g/hr) until case completion, per institutional protocol. In addition to routinely available packed red blood cells, platelets and DDAVP were ordered to be available in the operating room.

**Figure 2:**
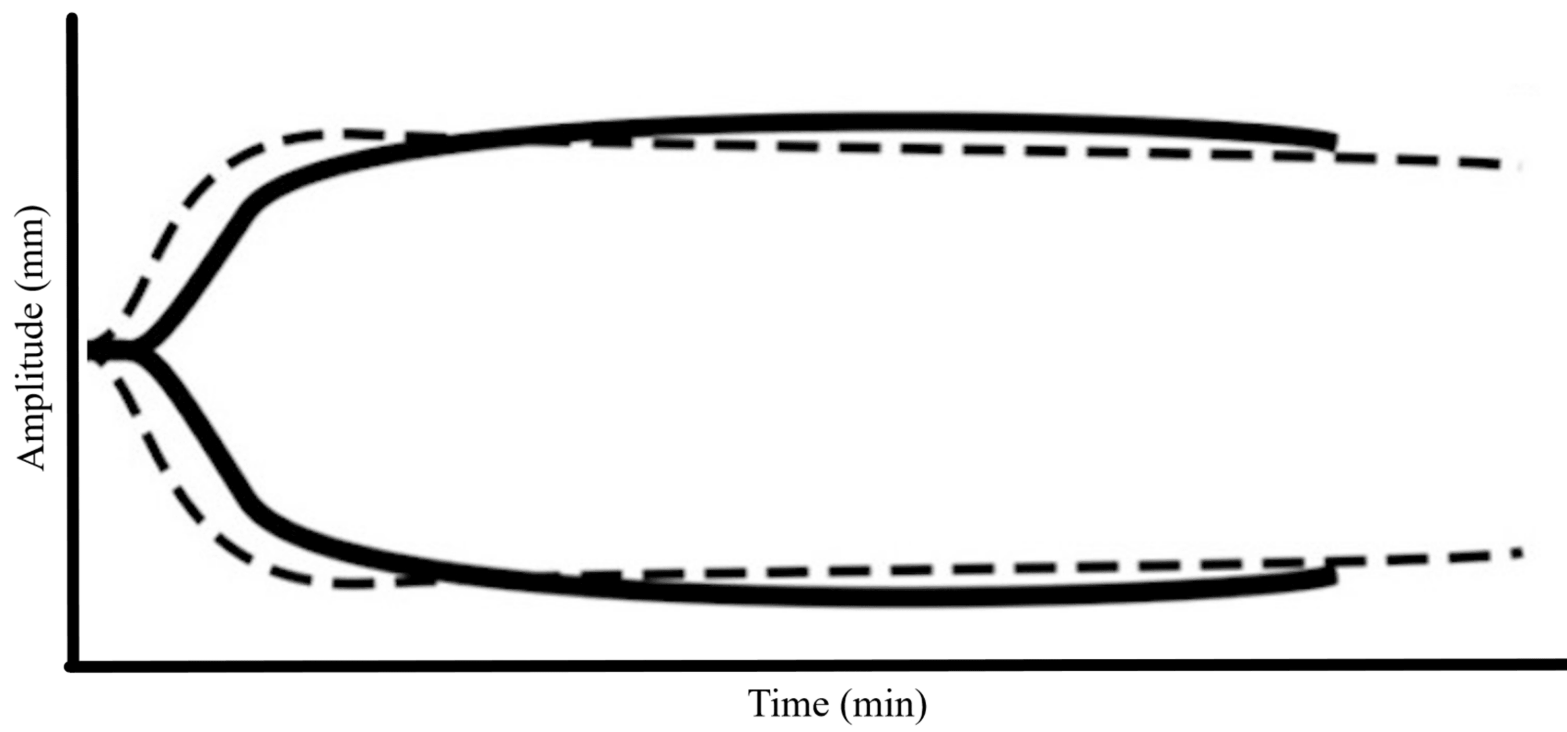
Standard TEG tracing 10 minutes after heparin reversal with protamine, showing no significant coagulopathy. The dashed line represents a normal reference TEG, while the solid line depicts the patient’s TEG. TEG: Thromboelastography®.

**Figure 3:**
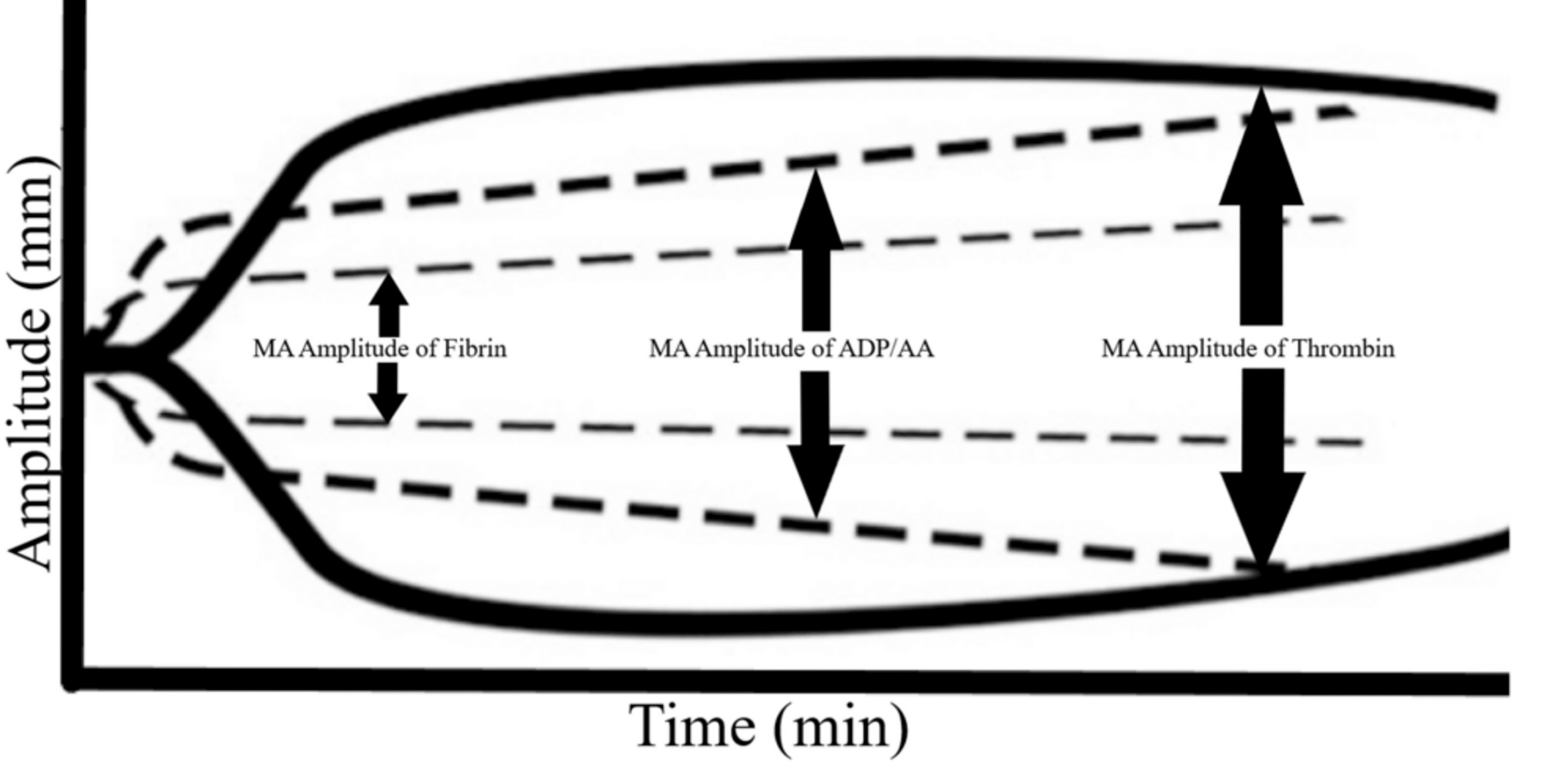
TEG with Platelet Mapping (TEG-PM) tracing 10 minutes after heparin reversal with protamine, demonstrating platelet inhibition. The solid line represents the standard TEG, indicating the MA amplitude of thrombin. MA Fibrin is also shown (small dashed line), along with platelet activation via ADP (large dashed line) and/or AA TEG: Thromboelastography; TEG-PM: Thromboelastography with Platelet Mapping; MA: Maximum Amplitude; ADP: Adenosine Diphosphate; AA: Arachidonic Acid.

The patient was heparinized with 20,000 IU to achieve an activated clotting time (ACT) >480 seconds, and cardiopulmonary bypass (CPB) was initiated without incident. Coronary artery bypass grafting was performed using a left internal mammary artery graft to the LAD and a saphenous vein graft to an obtuse marginal artery, while the RCA lesion was avoided due to poor targets. CPB time was one hour and thirty-one minutes. Protamine was administered for heparin reversal in standard fashion, and a repeat ACT confirmed return to the patient’s baseline. A standard TEG and TEG-PM were drawn. Following protamine administration, the surgeon requested a unit of platelets for a coagulopathic-appearing surgical field. Standard TEG at that time (Figure 2, Table 1) did not show significant derangements; however, TEG-PM demonstrated overall platelet inhibition of 38.9% for ADP (reference: 0-17%) and 91.6% for arachidonic acid (AA) (reference: 0-11%) (Figure 3, Table 2). Based on this information, the decision was made to administer DDAVP (0.3 mcg/kg) simultaneously with the unit of platelets. Hemostasis was achieved, and no further bleeding complications occurred. The patient also received 450 mL of cell salvage, and the surgical site was closed. She was transferred to the ICU and extubated after reversal of neuromuscular blockade. Postoperatively, no additional bleeding occurred, and the patient was discharged home in stable condition on postoperative day 5.

**Table 1:** Standard TEG results 10 minutes after heparin reversal with protamine, showing no significant coagulopathy. TEG®; Haemonetics, Braintree, MA, USA.

Standard TEG Parameters	Patient Values	Reference Range
R Time	7.4	5-10 min
K Time	1.7	1-3 min
Alpha Angle	67.3	53-72°
Maximum Amplitude (MA)	53.7	50-70 mm
G (Clot Strength)	5.8	5.3-12.4 dynes/cm²
Estimated Percent Lysis	0	0-15%
LY30 (Lysis at 30 Minutes)	0	0-8%
A30 (Amplitude at 30 Minutes)	53.7	50-70 mm

**Table 2:** TEG with Platelet Mapping (TEG-PM) results 10 minutes after heparin reversal with protamine, showing platelet inhibition. ((MA ADP/AA - MA Fibrin)/(MA Thrombin - MA Fibrin) x 100) = %Aggregation (100 - %Aggregation) = %Inhibition TEG®; Haemonetics, Braintree, MA, USA TEG: Thromboelastography; TEG-PM: Thromboelastography with Platelet Mapping; MA: Maximum Amplitude; ADP: Adenosine Diphosphate; AA: Arachidonic Acid

TEG-PM Parameters	ADP Patient Values (Reference Range)	AA Patient Values (Reference Range)
Maximum Amplitude (MA) of Thrombin	53.7 (53-68 mm)	53.7 (53-68 mm)
Maximum Amplitude (MA) of Fibrin	15.7 (2-19 mm)	15.7 (2-19 mm)
Maximum Amplitude (MA) of ADP/AA	38.9 (45-69 mm)	18.9 (51-71 mm)
Aggregation (%)	61 (83-100)	8.4 (89-100)
Inhibition (%)	38.9 (0-17)	91.6 (0-11)

## Discussion

MHA is classified as one of the four subtypes of inherited giant platelet disorders (IGPD), characterized by giant platelets (mean platelet volume >12.5 fL), thrombocytopenia, and inclusion bodies within leukocytes [1]. Despite the reduction in overall platelet count, platelet function in MHA generally remains intact, though complications can still occur, ranging from minor to severe bleeding [1]. Based on case reports and expert opinion, prophylactic administration of blood products prior to surgery is not suggested in MHA patients. Althaus and Greinacher, German physicians with expertise in transfusion medicine, do not recommend empiric perioperative transfusion, regardless of the degree of thrombocytopenia [6]. Instead, they suggest a perioperative protocol administering DDAVP (0.3 μg/kg) both preoperatively and postoperatively, along with oral tranexamic acid (0.5 g TID) for five days postoperatively [6]. They also emphasize the importance of withholding non-steroidal anti-inflammatory drugs (NSAIDs) and avoiding anemia, as a low circulating RBC volume can reduce primary hemostasis and worsen bleeding [6].

Both obstetric [2,7] and neurosurgical [3] cases have shown favorable outcomes without transfusion. A parturient with MHA underwent three cesarean sections; she received preoperative platelets during her first cesarean section but none during the subsequent two [2]. No bleeding complications were encountered perioperatively in any of the three surgeries, and all deliveries were uneventful [2]. As a result, the authors recommend against transfusion unless there are previously documented episodes of hemorrhage or active bleeding. Similarly, Fishman EB et al. managed three sisters with MHA during labor [7]. Each patient received neuraxial anesthesia, none received platelet transfusions at any time, and no bleeding occurred. Finally, a patient with MHA who developed cervical myelopathy underwent multi-level spine surgery. In this case, platelet transfusion was deferred despite profound thrombocytopenia of 0.6 × 10⁴/µL (150-400 × 10³/µL), as the functional assessment, including platelet aggregation, was preserved [3]. No bleeding issues were encountered postoperatively; however, the authors recommend considering TEG or similar technology to evaluate platelet function in patients undergoing surgery with a heightened risk of bleeding [3].

Extrapolating these surgeries to MHA patients undergoing cardiac surgery, with the combined deleterious effects of CPB-induced platelet dysfunction, remains challenging. In an emergent CABG, Everlien M et al. administered DDAVP prior to incision and transfused platelets during CPB in an MHA patient with a preoperative platelet count of 30 × 10³/µL (150-400 × 10³/µL) [4]. No bleeding complications or further transfusion occurred during or after the operation [4]. In our case, preoperative transfusion was deferred at a platelet level of 93 × 10³/µL (150-400 × 10³/µL), although DDAVP and platelets were made readily available intraoperatively. Upon separation from CPB and protamine reversal, standard TEG did not reveal any quantifiable factor deficiencies (Figure 2, Table 1). However, TEG-PM demonstrated platelet inhibition via ADP and AA (Figure 3, Table 2). We believe this was likely not attributable to the underlying MHA, as these patients often exhibit normal affinity for ADP [1], and CPB has been shown to reduce platelet aggregation via ADP, AA, and collagen [8-9]. As a result, we elected to administer both platelets and DDAVP. Hemostasis was achieved, and no further bleeding or transfusion requirements occurred in the postoperative period.

## Conclusions

Given the limited literature, along with our findings, we believe that perioperative transfusion may not be required for all MHA patients but should instead be considered on an individual, case-by-case basis. At a minimum, or during emergencies, laboratory assessments including hemoglobin, hematocrit, and platelet count should be reviewed in patients with known platelet disorders. In the context of elective surgery, a more thorough perioperative evaluation should serve as the cornerstone of the anesthetic plan, incorporating a history of bleeding complications, prior surgical history, and hematology records when feasible. Though not performed in our case, pre-procedural viscoelastic testing could also serve as a valuable baseline prior to the onset of potential coagulopathy. Surgery-specific considerations, including blood product availability, DDAVP, and antifibrinolytics, should also be addressed. While we acknowledge the limitations and limited generalizability of our single case report, intraoperative utilization of point-of-care viscoelastic testing can provide additional guidance for individualized transfusion management in patients with rare platelet disorders.
